# CO_2_ Biofixation and Growth Kinetics of *Chlorella vulgaris* and *Nannochloropsis gaditana*

**DOI:** 10.1007/s12010-016-2062-3

**Published:** 2016-04-06

**Authors:** Michał Adamczyk, Janusz Lasek, Agnieszka Skawińska

**Affiliations:** Institute for Chemical Processing of Coal, Zamkowa 1, 41-803 Zabrze, Poland

**Keywords:** CO_2_ biofixation, Green algae, *Chlorella vulgaris*, *Nannochloropsis gaditana*, Growth kinetics

## Abstract

CO_2_ biofixation was investigated using tubular bioreactors (15 and 1.5 l) either in the presence of green algae *Chlorella vulgaris* or *Nannochloropsis gaditana*. The cultivation was carried out in the following conditions: temperature of 25 °C, inlet-CO_2_ of 4 and 8 vol%, and artificial light enhancing photosynthesis. Higher biofixation were observed in 8 vol% CO_2_ concentration for both microalgae cultures than in 4 vol%. Characteristic process parameters such as productivity, CO_2_ fixation, and kinetic rate coefficient were determined and discussed. Simplified and advanced methods for determination of CO_2_ fixation were compared. In a simplified method, it is assumed that 1 kg of produced biomass equals 1.88 kg recycled CO_2_. Advance method is based on empirical results of the present study (formula with carbon content in biomass). It was observed that application of the simplified method can generate large errors, especially if the biomass contains a relatively low amount of carbon. *N. gaditana* is the recommended species for CO_2_ removal due to a high biofixation rate—more than 1.7 g/l/day. On day 10 of cultivation, the cell concentration was more than 1.7 × 10^7^ cells/ml. In the case of *C. vulgaris*, the maximal biofixation rate and cell concentration did not exceed 1.4 g/l/day and 1.3 × 10^7^ cells/ml, respectively.

**Electronic supplementary material:**

The online version of this article (doi:10.1007/s12010-016-2062-3) contains supplementary material, which is available to authorized users.

## Introduction

At present, there is still a debate going on about excessive CO_2_ emissions and their impact on climate change. It was calculated by Belbute and Pereira (2015) that CO_2_ emissions are projected to increase from 36,131 Mt in 2013 to almost 51,883 Mt in 2100, and it is about 52.9 % above 2010 levels. However, some other predictions are more pessimistic (e.g., OECD estimated the CO_2_ emission in 2050 will be as 70 % above the emission in 2010) [1]. It is estimated that the main sources of emissions due to human activities (so-called anthropogenic sources) are industrial processes, such as combustion from stationary chambers (power plants) and from transportation (engines of vehicles). According to International Energy Agency, in 2013, global CO_2_ emissions reached 32.2 GtCO_2_, an increase of 2.2 % over 2012 levels. Most of the CO_2_ anthropogenic emission (more than 46 %) came from coal combustion. Considering CO_2_ emission from sectors, 42 and 23 % of total value came from “electricity and heat” and “transport,” respectively. The rest of the CO_2_ emission was related to industry and other activities [2] Moreover, CO_2_ emissions from passenger cars were calculated as 8.7 % of global energy-related carbon dioxide emissions in 2013 [3]. Microalgae can grow 10–50 times faster than terrestrial plants [4]. The major difference between land plants and algae are the presence/absence of roots, shoots, and leaves that represent sinks for energy. As a result of faster growth rate, CO_2_ removal efficiency of microalgae is ten times higher than that of terrestrial plants [5] because microalgae can focus more of the captured solar energy on storage as high-energy-density lipids. In the process of photosynthesis, CO_2_ is converted into sugars, inter alia, with the use of energy derived from the compound called ATP (adenosine triphosphate 5) and with the participation of an enzyme (Rubisco-ribulose bisphosphate carboxylase oxygenase) in the Calvin Cycle [6]. Higher efficiency of photosynthesis means higher carbon dioxide consumption. In the case of land plants, this efficiency is about 1 %, because most of the energy does not reach chloroplasts. Energy reaching plant cells is lost due to non-absorbed wavelengths, reflected and dissipated light, energy dissipated as heat, photorespiration, and respiration [7]. Efficiency of photosynthesis of microalgae can range from 3 % (*Spirulina* sp.) to 20 % (*Chlorella* sp.). Moreover, microalgae can be used as feedstock for biofuel production due to high concentration of lipids in cells [8]. Application of microalgae can be noted as a reasonable and efficient way of biofixation of CO_2_ [6, 9–13].

Species of the genera *Chlorella*, *Scenedesmus, Spirulina, Nannochloropsis*, and *Chlorococcum* are characterized by rapid growth, tolerance to stress factors, and tolerance against high concentrations of CO_2_, which indicates its effective accumulation and utilization [14–16].

Representatives of both types, *N*annochloropsis *gaditana* and *Chlorella vulgaris*, are often subject of studies related to cultivation process; however, *N. gaditana* has not been particularly investigated regarding CO_2_ biofixation. This issue was preliminary underscored by Skawińska et al. [17]. An advanced analysis is presented in this paper.

Biofixation of CO_2_ in the presence of algae still needs detailed analysis. Initially, it was assumed in other studies that 1 kg of produced biomass equals 1.88 kg recycled carbon dioxide. However, this simplified method is not accurate. The second, advanced method is based on empirical results of the present study (formula with carbon content in biomass). It will be described more closely in the next section. It assumes that the content of carbon in algae biomass (after their cultivation process) should be taken into account for determination of CO_2_ biofixation [18, 19].

The aim of this work is to evaluate the CO_2_ biofixation and growth rate of microalgae: *C. vulgaris* and *N. gaditana*. CO_2_ biofixation was determined by applying a simplified and advanced method which takes into account carbon content in dry biomass of the algae after cultivation.

## Materials and Methods

### Stock Cultures and Chemicals

*Chlorella* genus cells are single cells, characterized by small size (2–10 μm diameter), spherical shape, and green color. Representatives of this type were found in fresh water (lakes, ponds) [20]. Species of *Nannochloropsis* genus are characterized by a spherical or cylindrical shape, exist as single cells with a diameter of 3–4 μm, and are found in salt water reservoirs [21].

Cultures of microalgae: *C. vulgaris* (freshwater species, strain No. CCAP 211/11D) and *N. gaditana* (marine species, strain No. CCMP 527) were provided by The University of Almeria (UAL), Spain. Cultures are identified and cataloged in *Culture Collection of Algae and Protozoa*, Scotland. Glass, water, and medium were sterilized at 200° (thermal incubator) to eliminate bacteria and fungi. Cultivation was carried out under sterile conditions (sterilization, antibacterial filters). Cultivation of freshwater species was carried out on BBM medium containing K_2_HPO_4_ 75 mg/l, KH_2_PO_4_ 175 mg/l, MgSO_4_ × 7H_2_O 75 mg/l, NaNO_3_ 250 mg/l, CaCl_2_ × 2H_2_O 25 mg/l, NaCl 25 mg/l, EDTA–Na_4_ 50 mg/l, KOH 31 mg/l, FeSO_4_ × 7H_2_O 5 mg/l, ZnSO_4_ × 7H_2_O 1.5 mg/l, and MnCl_2_ × 4H_2_O 0.2 mg/l. Cultivation of marine species was carried out on F/2 medium, containing NaNO_3_ 75 mg/l, NaH_2_PO_4_ × H_2_O 5 mg/l, Na_2_SiO_3_ × 9H_2_O 30 mg/l, FeCl_3_ × 6H_2_O 3.1 mg/l, Na_2_EDTA × 2H_2_O 4.3 mg/l, CuSO_4_ × 5H_2_O 10 mg/l, ZnSO_4_ × 7H_2_O 22 mg/l, CoCl_2_ × 6H_2_O 10 mg/l, and MnCl_2_ × 4H_2_O 180 mg/l.

### Evaluation of Photobioreactors and Parameters

The cultures were incubated in a reactor with a capacity of 15 l and plastic bottles with a capacity of 1.5 l. The cultivation conditions were as follows: pH of 7, temperature of 25 °C, photoperiod of 8 h, CO_2_ concentrations of 4 and 8 vol%, gas flow rate of 100 l/h, V/S ratio of 2.44 and 1.98 m for 15 and 1.5 l reactor, respectively, time of incubation of 10 days. It should be explained that comparison of results from different types of reactors is limited. The different reactors have inherently different hydrodynamic mixing, which dictates differential light exposure. Kobayashi et al. compared different types of reactors (80 L aquarium tanks, 1 or 3 L bioreactors, and 80 L hanging bags). They noticed the growth differences in the different types and sizes of cultures. It was explained by the difference of the light penetration and circulation [22]. In the present study, the size was different; however, V/S ratio and shape of reactors were comparable. The cultures were grown in distilled water in order to maintain sterile conditions. The results of experiments conducted in both photobioreactors could have been compared, because the ratio of volume to the surface of reactors (V/S) was similar, i.e., 2.44 and 1.98 for 15 and 1.5 l reactor, respectively. *Chlorella* species were grown in a reactor with 15 l capacity while *Nannochloropsis* species in a reactor with a capacity of 1.5 l. Both reactors were lit by artificial light—T5 lamps (Plant grow type, Blau, 4 × 39 W)—emitting white color light (6500 K). These lamps emit light from the blue color spectrum in the wavelength range of 410–460 nm and the red color of the spectrum in the wavelength range of 645 nm–670 nm. This type of lamps enables efficient performance of the process of photosynthesis. The reactors were also lit by daylight. All experiments were carried out at comparable irradiation conditions. It was estimated that artificial irradiation (W/m^2^) was more than two times higher than daylight irradiation.

Photoperiod was set at 8 h. The cultures were grown at two different concentrations of carbon dioxide, namely 4 and 8 %. Carbon dioxide from a pressurized cylinder was mixed with air pumped by a vacuum pump. Gases were mixed on a tee and then they were introduced into the culture. The concentration of carbon dioxide in the introduced gas was calibrated using a Sick type analyzer (measurement range of 0–40 vol%). The flow rate was 100 l/h.

The pH was determined using a pH meter type pH/Cond 340i WTW. The temperature was measured using a thermoelement coupled with the same pH meter. The pH was calibrated using 0.1 M sodium hydroxide solution. The temperature inside the reactor was maintained using an EHEIM aquarium heater with a power of 25 W. The pH level, temperature, and concentration of carbon dioxide in the inset gas were evaluated each day of the experiment. A sample of the culture (50 ml) was taken every day.

Drops of culture samples were taken and placed in a Marienfeld Thoma chamber. This chamber allows to determine the number of cells in 1 ml of culture. The Thoma chamber was placed under Olympus light microscope at ×400 magnification. The number of cells was counted in 60 small squares of Thoma chamber and an average number of cells was counted per small square. This value was substituted into the formula *D* = *a* × *b* × 4000 × 1000 where *D* is the number of cells in 1 ml, *a* the average number of cells in one small square of Thoma chamber, and *b* the dilution of the culture.

Subsequently, 50 ml of the culture was placed in a centrifuge—MPW 260R Centrifuge. The sample was centrifuged for 30 min at 4 °C at a rotation speed equal to 5000 rev/min. Centrifugation enabled separation of the biomass of microalgae culture with over 97 % efficiency. The supernatant was decanted and the obtained biomass was dried in a thermal test chamber—WAMED 65 W at 70 °C. After drying, the weight of biomass was measured on Sartortius–Secura balance (measurement range of 0.0100 to 220.0000 g, inaccuracy of 0.0001 g) to determine the concentration of biomass in culture (g l^−1^). Knowing the concentration of biomass, productivity (*P*, g l^−1^ day^−1^) and biofixation of carbon dioxide were measured using Eq. (1):

1 $$ {\mathrm{CO}}_2\mathrm{biofixation}=C\times P\times \left({\mathrm{MCO}}_2/\mathrm{M}\mathrm{C}\right) $$

where the symbols stand for *C*—carbon content in the biomass, *P*—productivity, MCO_2_—molar mass of carbon dioxide, and MC—molar mass of carbon. This method of determination of CO_2_ biofixation is advanced compared to the simplified method. The simplified method assumes that 1 kg of produced biomass equals 1.88 kg recycled carbon dioxide [23, 24].

The carbon content in biomass was determined using an elemental analyzer: Elementar VarioMacroCube CHNS. After 10 days, a part of the cultivation was frozen as an inoculum for the next culture.

## Results

### Biomass Concentration and Productivity

In order to determine the carbon dioxide biofixation by freshwater and marine microalgae, the productivity was calculated. Productivity is the increase of the weight of biomass per time unit. This parameter was determined by measuring the weight of biomass contained in the volume unit. The above-mentioned parameters were determined each day of the cultivation under the same conditions. Results of the experimental concentration and productivity of species *C. vulgaris* are shown in Table 1. *N. gaditana* species are presented in Table 2.

**Table 1:** Concentration and productivity of a cultivation of *Chlorella vulgaris* species at CO_2_ concentrations of 4 and 8 %

Day of cultivation		1	2	3	4	5	6	7	8	9	10
CO_2_ concentration		4 %									
Concentration, g/l	Value	0.4	0.6	0.7	0.8	1.3	1.81	2.58	2.89	3	3.15
	±	0.006	0.008	0.009	0.01	0.02	0.02	0.028	0.03	0.03	0.03
Productivity, g/l/day	Value	0	0.2	0.1	0.1	0.5	0.51	0.77	0.31	0.11	0.15
	±		0.014	0.017	0.019	0.02	0.04	0.05	0.06	0.06	0.07
CO_2_ concentration		8 %									
Concentration, g/l	Value	0.33	0.93	1.13	1.55	1.68	2.2	2.56	3.12	3.25	3.33
	±	0.005	0.011	0.013	0.017	0.019	0.024	0.028	0.03	0.03	0.04
Productivity, g/l/day	Value	0	0.6	0.19	0.4	0.16	0.56	0.36	0.56	0.13	0.08
	±		0.017	0.025	0.03	0.04	0.04	0.05	0.06	0.07	0.07

**Table 2:** Concentration and productivity of cultivation of *Nannochloropsis gaditana* species at CO_2_ concentrations of 4 and 8 %

Day of cultivation		1	2	3	4	5	6	7	8	9	10
CO_2_ concentration		4 %									
Concentration, g/l	Value	0.55	0.58	0.8	1.7	2.2	2.5	3.44	3.92	3.98	4.05
	±	0.36	0.34	0.25	0.12	0.09	0.08	0.06	0.05	0.05	0.05
Productivity, g/l/day	Value	0	0.03	0.22	0.9	0.5	0.3	0.94	0.48	0.06	0.07
	±		0.015	0.018	0.029	0.04	0.05	0.06	0.08	0.08	0.08
CO_2_ concentration		8 %									
Concentration, g/l	Value	0.24	0.54	0.63	1.0	1.5	2.34	3.44	3.76	3.96	4.02
	±	0.8	0.4	0.3	0.20	0.13	0.09	0.06	0.05	0.05	0.05
Productivity, g/l/day	Value	0	0.3	0.09	0.37	0.5	0.84	1.1	0.32	0.2	0.06
	±		0.012	0.016	0.020	0.029	0.042	0.06	0.08	0.08	0.08

Biomass concentration in both algal cultures of *C. vulgaris* was increasing monotonically along with the duration of the cultivation and reached the maximum level on the last day of cultivation. In both cultures, concentration reached a value of above 3 g/l. The increments of biomass were different between the cultures of this species. In the culture with the concentration of carbon dioxide of 4 %, productivity was gradually increased to reach the maximum value in the middle of the culture period and then it was gradually decreased. A similar dependence in the culture with a concentration of carbon dioxide up to 8 % was not observed. Biomass increments had similar values both at the beginning of the culture and at its end.

In the cultivations of the marine species *N. gaditana*, concentration of biomass in both cultures exceeded 4 g/l on the last day of cultivation (see Table 2). These values are significantly higher than the concentration of biomass in *Chlorella* species. A higher concentration of biomass gives a possibility of obtaining not only a larger quantity of biomass with the same volume of culture but also an increase of the carbon dioxide biofixation. The largest increase of biomass per unit of time, both in the culture with 4 % carbon dioxide concentration and with 8 %, was observed after about 5 days from the start of cultivation. In the culture with a higher concentration of carbon dioxide (8 %), productivity exceeded 1 g/l/day.

### Biofixation

Biofixation of carbon dioxide was calculated in two ways. At first, using a simplified method (M1), it was assumed that 1 kg of produced biomass equals 1.88 kg recycled carbon dioxide (see M1 values in Table 3) [23]. As a result, the amount of carbon dioxide recycled in the cultures of microalgae species was calculated. In the case of cultures of *Chlorella* species, the total amount of recycled carbon dioxide during 10 days culture was 5.1 and 5.2 gCO_2_/l for CO_2_ concentrations of 4 and 8 %, respectively. In the case of cultures of *Nannochloropsis* species, the total amount of recycled carbon dioxide was higher, namely 6.3 and 6.9 gCO_2_/l for CO_2_ concentrations of 4 and 8 %, respectively (see Table 3).

**Table 3:** Biofixation of carbon dioxide in cultures of *Chlorella vulgaris* and *Nannochloropsis gaditana* calculated using two methods (M1, M2)

*Chlorella vulgaris*				*Nannochloropsis gaditana*			
M1 (gCO2/l/d)		M2 (gCO2/l/d)		M1 (gCO2/l/d)		M2 (gCO2/l/d)	
4 %	8 %	4 %	8 %	4 %	8 %	4 %	8 %
0	0	0.00	0.00	0	0	0.00	0.00
0.4	1.1	0.18	0.55	0.1	0.5	0.05	0.48
0.2	0.3	0.09	0.17	0.4	0.2	0.35	0.15
0.2	0.7	0.09	0.37	1.6	0.7	1.45	0.60
0.9	0.3	0.46	0.15	0.9	0.9	0.81	0.81
0.9	0.9	0.47	0.48	0.5	1.5	0.48	1.36
1.4	0.6	0.71	0.33	1.7	2	1.52	1.77
0.6	1	0.28	0.51	0.9	0.6	0.77	0.50
0.2	0.2	0.10	0.12	0.1	0.4	0.10	0.32
0.3	0.1	0.14	0.07	0.1	0.1	0.11	0.10
5.1	5.2	2.52	2.75	6.3	6.9	5.65	6.08

**Fig. 1:**
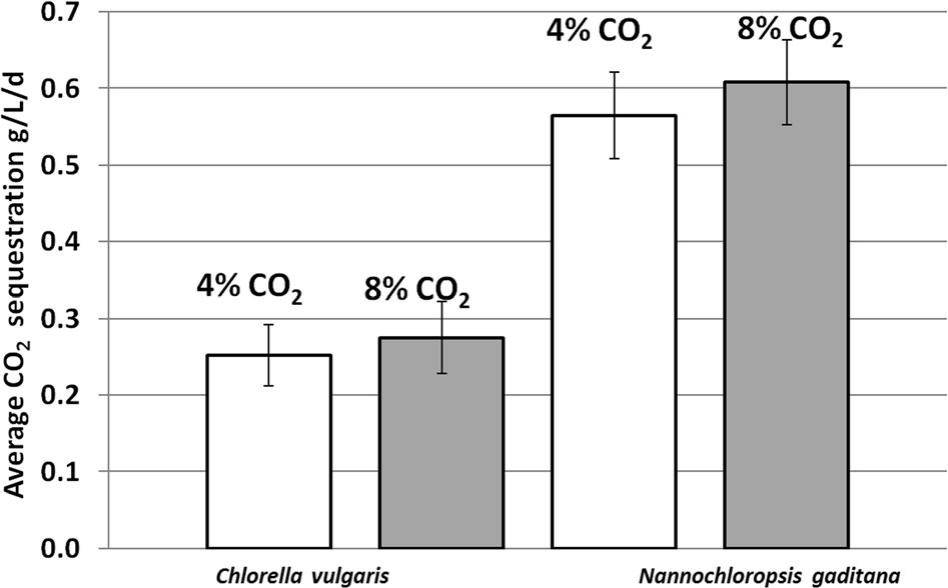
Average amount of recycled carbon dioxide in cultures of *Chlorella vulgaris* and *Nannochloropsis gaditana*

The first method of calculating the amount of recycled carbon dioxide is affected by a significant error [19]. Therefore, the second method applied assumes the use of Eq. (1) [18]. This allowed for a more accurate calculation of CO_2_ biofixation. For all cultures, the M2 values were shown in Table 3. The results of the two calculation methods were significantly different only in freshwater species. Values of M2 were twice as high compared to the values obtained using the simplified method. In the advanced method (see M2 values in Table 3), the percentage of carbon in the biomass was estimated using CHNS elemental analyzer. The percentages (mass fractions) of carbon, nitrogen, hydrogen, and sulfur content of the dried biomass of *C. vulgaris* species were, respectively, C = 25 %, N = 10.6 %, H = 4.5 %, and S = 2.5 %. Results for *N. gaditana* were C = 44 %, N = 9.8 %, H = 6.9 %, and S = 0.6 %. The rest of each sample were oxygen and ash. These values were used in Eq. (1). Considering *N. gaditana* species, the difference in biofixation value (calculated by simplified and advanced method) was not so high; however, in the case of *C. vulgaris*, the differences were dramatically large. The difference in biofixation between species was also significant. *N. gaditana* assimilated twice as much carbon dioxide as *C. vulgaris*, as shown in Fig. 1. It was also observed that the amount of recycled carbon dioxide was higher in cultures of both species with carbon dioxide at a concentration of 8 %. Sulfur, nitrogen, and hydrogen content in the biomass were measured using CHNS elemental analyzer.

### Compaction of Culture

**Fig. 2:**
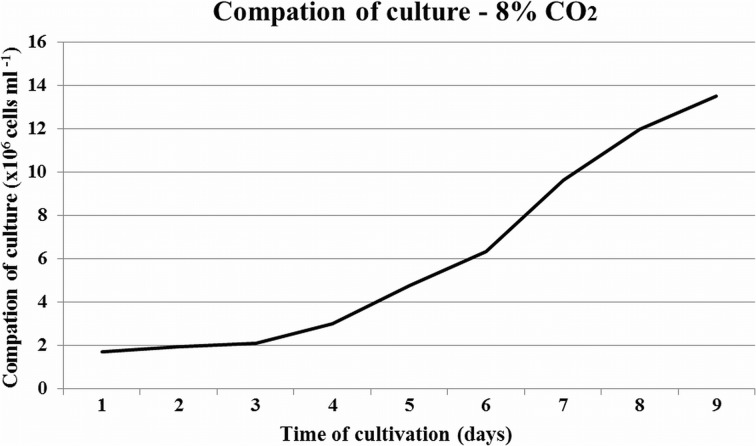
Compaction of *Chlorella vulgaris* culture during 10 days of cultivation

The cell concentration was calculated each day of conducted cultivation. Example of measurements of culture compaction for *C. vulgaris* are presented in Fig. 2. Number of cells per 1 ml of culture increased during cultivation. In all cultures, the number of cells on the final day of cultivation exceeded 10^6^ cells/ml. In the case of *C. vulgaris* on day 10 of cultivation, the cell concentration was 1.2 × 10^7^ and 1.3 × 10^7^ cells/ml for CO_2_ concentrations of 4 and 8 vol%, respectively. In the case of *N. gaditana* species, a large number of cells was observed. On day 10 of cultivation, the cell concentration was 1.8 × 10^7^ and 1.7 × 10^7^ cells/ml for CO_2_ concentrations of 4 and 8 vol%, respectively. This is due to the size of the microalgae. Cells of the saltwater species *N. gaditana* are significantly smaller than those of the freshwater species *C. vulgaris* (see Fig. 2).

## Discussion

### Biofixation

The microalgae chemical composition should be discussed. Redfield mentioned that “The formation of organic matter in the autotrophic zone requires all the elements in protoplasm, of which carbon, nitrogen, and phosphorus are of particular concern” [25]. Based on Fleming work [26], Redfield concluded that the ratio of carbon, nitrogen, and phosphorus in the plankton was C/N/P = 106:16:1. Redfield noticed that also some other elements (e.g., sulfur, calcium, magnesium, potassium) existed in the plankton, but these elements were not included in the empirical ratio. In the present work, microalgae composition was estimated as ultimate analysis of solid fuels. The obtained results are consistent with the results reported by other researchers [27–30]. However, the carbon content needs to be discussed. Typical carbon content (i.e., 46–51 w%) [27–30] in *C. vulgaris* is higher than the value obtained in this research (25 wt%). This analysis was double-checked to eliminate some measurement error. After this procedure, the same value was obtained. In fact, such low value is acceptable. For example, Kim et al. found that carbon content in dry *Sargassum* sp. was 26.7 wt% [31]. Differences between the cultures could also be attributed to pH drift (caused by CO_2_ concentrations) or nitrogen depletion, which could indeed vary between the two species and different CO_2_ culture conditions [32].

It should be underscored that the two methods for the determination of CO_2_ sequestering gave different results. The method which assumed that 1 g of produced biomass equals 1.8 g recycled CO_2_ is imprecise [19, 23]. The second method using the following parameters: the percentage of carbon in biomass, molecular weight of C and CO_2_, and the productivity, is a much more precise method [18]. Estimation of the amount of CO_2_ captured, calculating using Eq. (1), depends on the carbon content in the biomass. Carbon in the biomass of *Chlorella* species accounted for 25 % of total biomass; therefore, the calculated amount of CO_2_ captured (using the second method [2]) was significantly lower than in *Nannochloropsis* species. Additionally, considering *Chlorella* species, biofixation calculated using the simplified method was almost twice as high as in the case of calculation based on the advanced method (according to Eq. (1)). The maximum daily utilization of CO_2_ (calculated from M2) amounted to 0.71 g/l/day in 4 % CO_2_ culture of *Chlorella* species and 1.77 g/l/day for *Nannochloropsis* species in the culture of CO_2_ concentration of 8 %. Higher productivity means higher rate of biomass growth, and it results in a larger amount of sequestrated carbon dioxide. Higher biofixation was observed in the initial and middle stages of cultivations. Freshwater species of *C. vulgaris* is characterized by rapid growth. Therefore, *Chlorella* needs a large amount of carbon derived from carbon dioxide to grow. These microalgae utilized carbon dioxide in the range of 0.1–0.7 gCO_2_/l/day. At suitably selected optimal conditions during culture of this species is capable of utilizing 1 gCO_2_/l/day [24]. According to Ho et al. *C. vulgaris* is able to utilize more than 6 gCO_2_/l/day in a membrane-type reactor [33]. The advanced method of CO_2_ biofixation was used by Alhamed et al. [34]. They investigated CO_2_ biofixation at *C. vulgaris* species. The maximum fixation rate was found to be 0.415 gCO_2_/l/day. *N. gaditana* microalgae have not been studied (regarding CO_2_ fixation) extensively. In cultivations of similar conditions as in the case of *Chlorella* species, CO_2_ can be captured more effectively in the range of 0.1 to 1.8 gCO_2_/l/day. Table 4 shows a comparison of CO_2_ fixation in the presence of different algae species. This literature data reviewed by Wang et al. [24] and Ho et al. [33] is compared with the results obtained in this research. It can be noticed that *N. gaditana* is able to fix a large amount of CO_2_ compared to other species, including *C. vulgaris*. Moreover, it has been mentioned earlier that the amount of sulfur in the case of *C. vulgaris* is more than four times higher compared to *Nannochloropsis* biomass. Sulfur content is a crucial parameter if the biomass after cultivation is dedicated to fuel processing, e.g., biodiesel. Higher amount of sulfur in the biomass is not desired if algae are considered as feedstock for biofuel production.

**Table 4:** Comparison of CO_2_ biofixation (maximum value) by different algae species

Microalgae	CO_2_ fraction in inlet gas, vol%	*T*, °C	CO_2_ fix., g/l/day
Reviewed by Wang et al. [24]			
* Chlorococcum littorale*	40	30	1.0
* Chlorella kessleri*	18	30	0.163^a^
* Chlorella vulgaris*	15	–	0.625
* Chlorella vulgaris*	Air	25	0.075^a^
* Chlorella sp*.	40	42	1.0
* Dunaliella*	3	27	0.313^a^
* Haematococcus pluvialis*	16–34	20	0.143
* Scenedesmus obliquus*	Air	–	0.031
* Botryococcus braunii*	–	25–30	>1.0
* Scenedesmus obliquus*	18	30	0.26
* Spirulina sp*.	12	30	0.413^a^
Reviewed by Ho et al. [33]			
* Anabena sp*.	Air	–	1.450
* Nannochloropsis sp*.	15	–	0.601
* Phaeodactylum tricornutum*	15	–	0.282
This research (calculated from M2)			
* Chlorella vulgaris*	4	25	0.71
* Chlorella vulgaris*	8	25	0.55
* Nannochloropsis gaditana*	4	25	1.52
* Nannochloropsis gaditana*	8	25	1.77

^a^Calculated using simplified method

Biofixation of carbon dioxide depends on a variety of factors used in cultivation. CO_2_ removal efficiency is mainly affected by the following parameters: CO_2_ concentration in the culture, microalgae species, temperature, pH, and photoperiod but also, to a lesser extent, shape and size of the reactor [22, 35] as well as initial biomass concentration [34]. The impact of each listed parameter is different. It is known that effective utilization of CO_2_ depends mainly on the type of species and concentration of CO_2_ introduced into the culture [36]. There are no ideal microalgae species for all applications. Some species are desirable for biofuel production due to high lipid content in the cells; others are characterized by a very rapid growth of biomass. In the future, the key to effective application of microalgae in different industries can be the use of genetic engineering. This will allow to obtain species-linking properties of both rapid growth and high lipid content in the cells [37]. Many species of microalgae are able to survive even if they are exposed to high concentrations of CO_2_. However, the appropriate concentration in the culture, selected for the particular species, allows for optimal growth. Microalgae grow best at a concentration of CO_2_ in the range of 4–12 %. The differences in efficiency of biofixation in species of *Chlorella* and *Nannochloropsis* were also dependent on the concentration used. In our study, the difference in cultures with carbon dioxide concentrations of 4 and 8 % was negligible. Tang et al. [38] investigated CO_2_ fixation using *Chlorella pyrenoidosa* and *Scenedesmus obliquus* species. The CO_2_ concentration was varied in the range of 0.03–50 vol%. They reported that optimal concentration of CO_2_ for efficient fixation was 10 vol%; however, the researchers noticed slight differences in CO_2_ fixation for CO_2_ concentration in the range of 5–10 vol%. A maximal CO_2_ fixation rate was 0.288 gCO_2_/l/day for *S. obliquus*.

Kumar et al. [39] reported that CO_2_ fixation in the presence of *Chlorella sorokiniana* species equaled more than 3 gCO_2_/l/day. However, it should be noticed that CO_2_ biofixation is only one of the many possibilities of microalgae application [39]. *C. vulgaris* species contains valuable micro- and macroelements beyond biofixation that can be used for other applications. *N. gaditana* species has a high lipid content so it can be used for production of biofuels [40, 41]. Apart from CO_2_ fixation, microalgae cultivation can be used for the production of cosmetics, supplements, biogas, bioethanol, and other biofuels.

### Compaction of Culture

Illman et al. [42] has appointed cell concentration of several species of the *Chlorella* genus. Cell concentration was determined using a hemocytometer. This equipment is very similar to the Thoma chamber. The smallest density was indicated by a species of *Chlorella protothecoides*, namely 0.5 × 10^6^ cell/ml. The highest density was reached by *Chlorella emersonii*, 6.5 × 10^6^ cells/ml. These values were different between species, depending on the culture conditions (e.g., nitrogen concentration). *N. gaditana* was investigated by Rocha et al. [43]. After 14 days of cultivation, the maximum density was 5.5 × 10^7^ cells/ml. In our study, the density was estimated above 10^6^ cells/ml. It resulted from rapid growth of both species, using lamps supporting growth and using optimal medium for growth. Assuming sphericity and average cell size, cells surface per unit weight can be estimated. Specific surface area (per culture mass) can be useful for bioreaction modeling including CO_2_ biofixation.

### Kinetics of Growth

The kinetics of growth of microalgae can be considered taking into account the variability of different culture parameters. Béchet et al [44] analyzed the kinetics of growth of microalgae models taking into account the intensity of light, temperature, and other process parameters. To analyze the growth rate, a model used recently by Kumar et al. [39] was applied. The differential form of the model is described by Eq. (2):

2 $$ \frac{dC}{dt}={K}_CC\left(1-\frac{C}{C_{\max }}\right) $$

The integral form of the kinetic equation is

3 $$ C=\frac{C_{\max }}{1+\left(\frac{C_{\max }}{C_0}-1\right){e}^{-{K}_Ct}} $$

where the parameters are *C*—concentration of algae in the cultivation period, gl^−1^; *t*—time, day; *K*_c_—growth rate factor, day^−1^; *C*_max_—maximum concentration, gl^−1^; and *C*_0_—the initial value of the concentration, gl^−1^. Designated coefficient parameters *K*_c_ for each species and participation of CO_2_ in the gases fed to the reactor are shown in Table 5. MAE, %, is the average absolute error (mean absolute error, MAE), defined as [45]:

4 $$ \mathrm{M}\mathrm{A}\mathrm{E}=\frac{1}{n}{\displaystyle {\sum}_{i=1}^n\left|\frac{C_{\mathrm{predicted}}-{C}_{\mathrm{experimental}}}{C_{\mathrm{experimentak}}}\right|}\times 100\% $$

where *C*_predicted_ and *C*_experimental_ mean, respectively, the concentration calculated by the model and experimentally determined values.

**Table 5:** Model coefficients of growth rate of *Chlorella vulgaris* and *Nannochloropsis gaditana* microalgae

Algae	CO_2_, %	*K*_c_, day^−1^	MAE, %
*Chlorella vulgaris*	4	0.41	28
*Chlorella vulgaris*	8	0.51	12
*Nannochloropsis gaditana*	4	0.44	29
*Nannochloropsis gaditana*	8	0.45	20

**Fig. 3:**
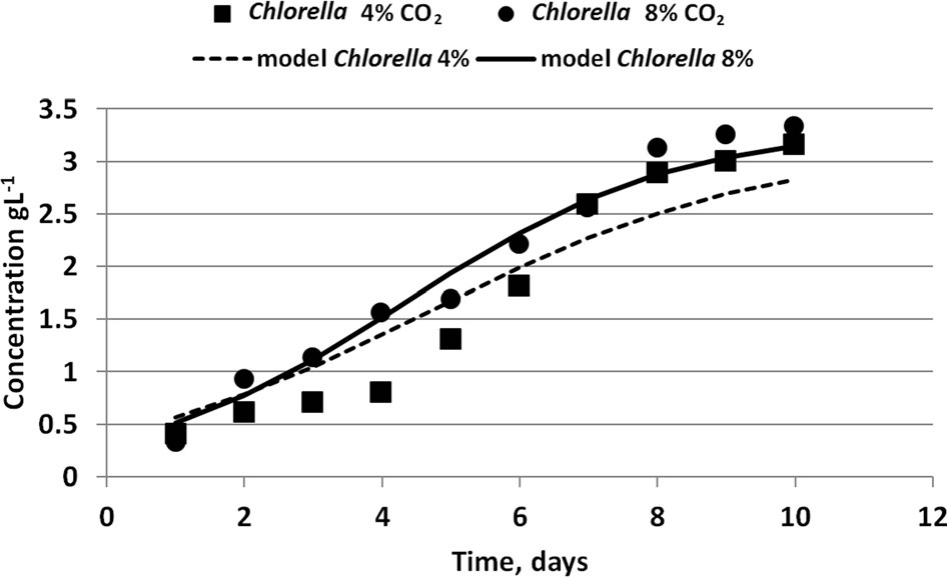
Comparison of concentration values determined experimentally and by Eq. (3) for *Chlorella vulgaris* species

Table 5 shows determined kinetic coefficients of growth rate of *C. vulgaris and N. gaditana* species. A comparison of concentration values obtained experimentally and by Eq. (3) for the species *C. vulgaris* is shown in Fig. 3.

Growth rate coefficients determined by Kumar et al. [39] refer to *C. sorokiniana* species. The values of *K*_c_ coefficient obtained by these researchers were 0.4 per day, which are similar to those presented in our study. However, it should be noticed that this *K*_c_ was obtained by Kumar et al. where the bioreactor was fed with flue gas. When the flue gas was diluted by air (air-flue proportion of 3:1) the *K*_c_ coefficient was 1.3 per day.

## Conclusions

The subject of the study was CO_2_ biofixation in the presence of *C. vulgaris* and *N. gaditana*. CO_2_ biofixation was determined using a simplified and an advanced method that consists in carbon analysis in biomass. It was observed that application of the simplified method can generate large errors, especially if the biomass contains a relatively low amount of carbon. Thus, it is recommended to use the advanced method to determine CO_2_ fixation.

*N. gaditana* species is characterized by higher CO_2_ fixation rate (average more than 0.55 g/l/day) compared to the reference species, i.e., *C. vulgaris*; however, differences between the values of growth rate coefficient *K*_c_ for both species are not so high. *N. gaditana* species are recommended for CO_2_ capture process due to high CO_2_ fixation rate—more than 1.7 g/l/day. On day 10 of cultivation, the cell concentration was more than 1.7 × 10^7^ cells/ml. In the case of *C. vulgaris*, the maximal biofixation rate and cell concentration did not exceed 1.4 g/l/day and 1.3 × 10^7^ cells/ml, respectively.

## Electronic supplementary material

Below is the link to the electronic supplementary material.

Fig. S1 (GIF 56 kb)

High Resolution Image (TIF 550 kb)

Fig. S2 (GIF 144 kb)

High Resolution Image (TIF 2113 kb)
