# Supplementary figures and images for: CO2 Biofixation and Growth Kinetics of Chlorella vulgaris and Nannochloropsis gaditana
Source: Appl Biochem Biotechnol. 2016 Apr 6;179:1248–61. doi: 10.1007/s12010-016-2062-3 (PMC4978769; doi:10.1007/s12010-016-2062-3)

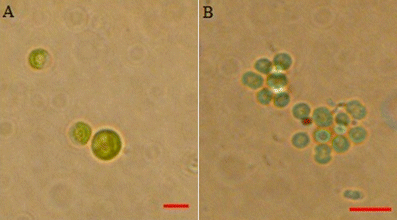

Supplement: Supplementary file 1 — (GIF 56 kb) [file 12010_2016_2062_Fig4_ESM.gif]

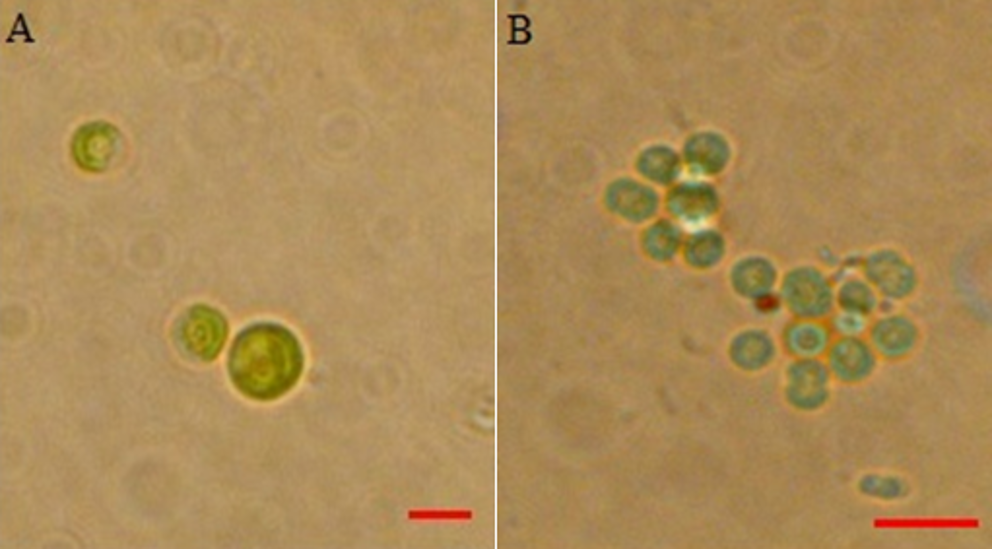

Supplement: Supplementary file 2 — High Resolution Image (TIF 550 kb) [file 12010_2016_2062_MOESM1_ESM.tif]

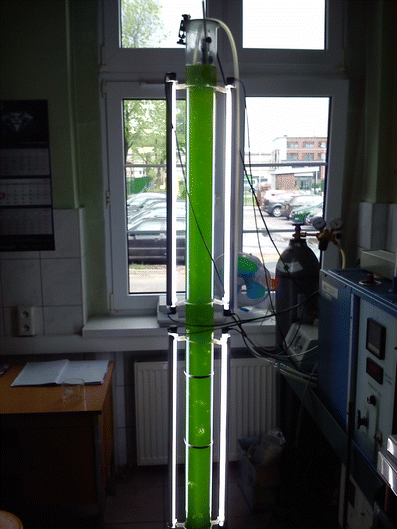

Supplement: Supplementary file 3 — (GIF 144 kb) [file 12010_2016_2062_Fig5_ESM.gif]

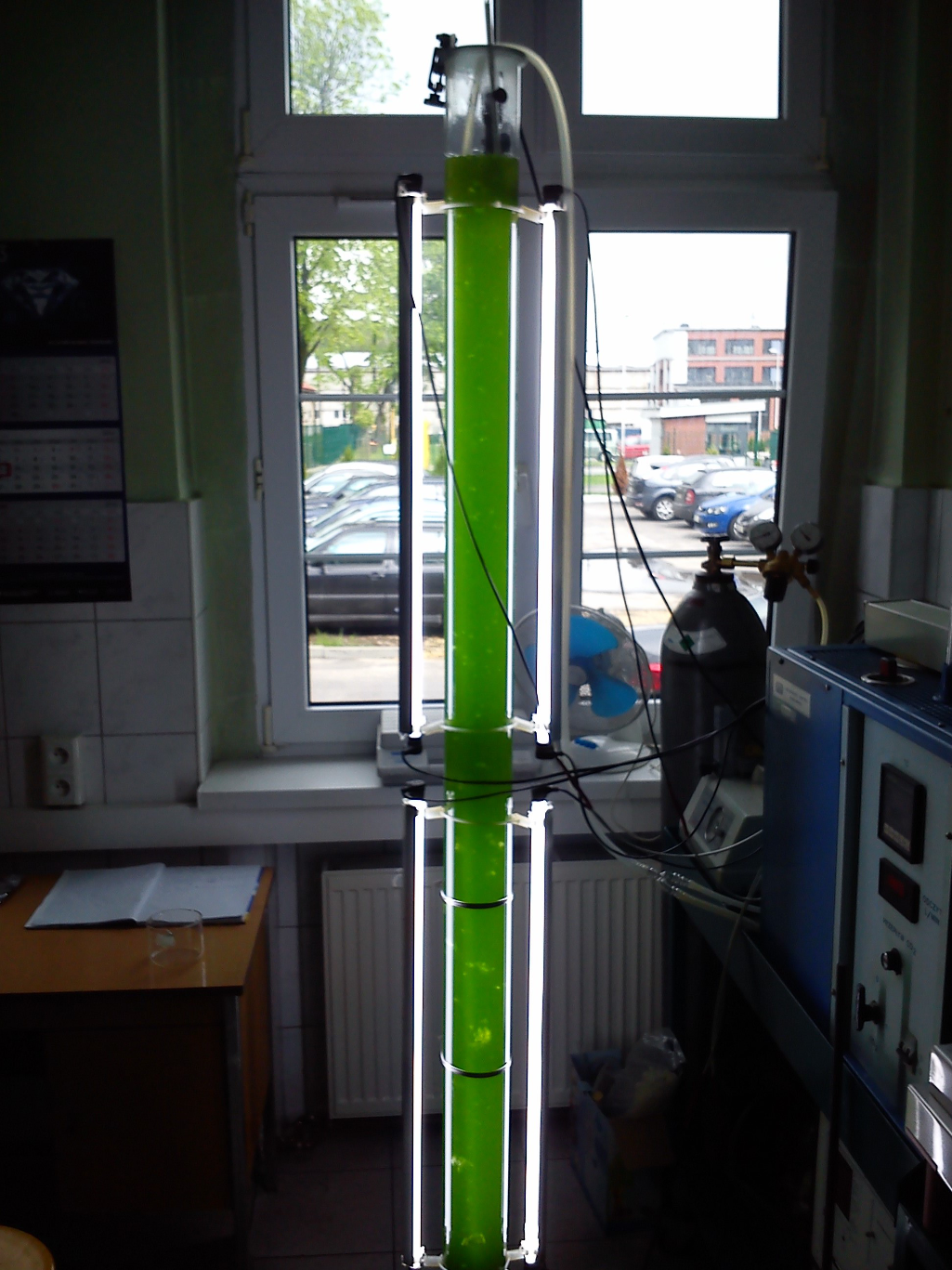

Supplement: Supplementary file 4 — High Resolution Image (TIF 2113 kb) [file 12010_2016_2062_MOESM2_ESM.tif]
